# Inhibition of the Unfolded Protein Response Mechanism Prevents Cardiac Fibrosis

**DOI:** 10.1371/journal.pone.0159682

**Published:** 2016-07-21

**Authors:** Jody Groenendyk, Dukgyu Lee, Joanna Jung, Jason R. B. Dyck, Gary D. Lopaschuk, Luis B. Agellon, Marek Michalak

**Affiliations:** 1 Department of Biochemistry, University of Alberta, Edmonton, Alberta, T6G 2H7, Canada; 2 School of Dietetics and Human Nutrition, McGill University, Ste. Anne de Bellevue, Quebec, H9X 3V9, Canada; 3 Department of Pediatrics, University of Alberta, Edmonton, Alberta, T6G 2H7, Canada; 4 Department of Pharmacology, University of Alberta, Edmonton, Alberta, T6G 2H7, Canada; University of Hong Kong, HONG KONG

## Abstract

**Background:**

Cardiac fibrosis attributed to excessive deposition of extracellular matrix proteins is a major cause of heart failure and death. Cardiac fibrosis is extremely difficult and challenging to treat in a clinical setting due to lack of understanding of molecular mechanisms leading to cardiac fibrosis and effective anti-fibrotic therapies. The objective in this study was to examine whether unfolded protein response (UPR) pathway mediates cardiac fibrosis and whether a pharmacological intervention to modulate UPR can prevent cardiac fibrosis and preserve heart function.

**Methodology/Principal Findings:**

We demonstrate here that the mechanism leading to development of fibrosis in a mouse with increased expression of calreticulin, a model of heart failure, stems from impairment of endoplasmic reticulum (ER) homeostasis, transient activation of the unfolded protein response (UPR) pathway and stimulation of the TGFβ1/Smad2/3 signaling pathway. Remarkably, sustained pharmacologic inhibition of the UPR pathway by tauroursodeoxycholic acid (TUDCA) is sufficient to prevent cardiac fibrosis, and improved exercise tolerance.

**Conclusions:**

We show that the mechanism leading to development of fibrosis in a mouse model of heart failure stems from transient activation of UPR pathway leading to persistent remodelling of cardiac tissue. Blocking the activation of the transiently activated UPR pathway by TUDCA prevented cardiac fibrosis, and improved prognosis. These findings offer a window for additional interventions that can preserve heart function.

## Introduction

The endoplasmic reticulum (ER) is a multifunctional organelle responsible for many cellular housekeeping functions including proteins synthesis, lipid synthesis, storage and release of Ca^2+^, and regulation of gene expression and energy metabolism [1, 2]. Disrupted ER homeostasis leads to activation of ER stress coping responses and appropriate corrective strategies including activation of the unfolded protein response (UPR) [3, 4]. Activation of ER stress and UPR coping response has been associated with cardiac pathology and heart failure [2, 5]. In cardiomyocytes, a highly specialized version of the ER is a critical component of excitation-contraction coupling [6]. The ER is highly sensitive to homeostatic changes and cellular stresses.

Cardiac fibrosis is a common final pathway for cardiac failure and a crucial determinant of myocardial heterogeneity and the propensity for re-entry arrhythmias [7–14]. It is characterized by excessive deposition of extracellular matrix (ECM) proteins in the myocardium and results in progressive organ dysfunction [15]. Extensive fibrotic remodelling of the ventricle is associated with acute myocardial infarction [16], pressure overload, diabetes [17], or obesity [18]. Cardiac fibrosis is difficult to treat in the clinic due to lack of effective anti-fibrotic therapies. Increased abundance of calreticulin, an ER Ca^2+^-buffering chaperone, is associated with human heart failure [19] and is mechanistically linked to the induction of cardiac hypertrophy [20–23]. There is a strong correlation between calreticulin overexpression and high prevalence of atrial fibrosis in patients with dilated cardiomyopathy [24, 25]. Indeed, overexpression of calreticulin in adult mouse hearts results in dilated cardiomyopathy with compromised systolic and diastolic function and heart failure [26]. These findings indicate that ER homeostasis is critical to cardiovascular pathophysiology [2]. Loss of ER homeostasis causes the activation of ER stress coping response pathways, which includes the unfolded protein response (UPR). We demonstrate here that mice with calreticulin overexpression, which were used to model heart failure, developed extensive cardiac fibrosis due to transient activation of UPR which in turn underlies the subsequent stimulation of the TGFβ1/Smad2/3 signaling pathway. Early inhibition of the IRE1α pathway of UPR in the heart by tauroursodeoxycholic acid (TUDCA) prevented cardiac fibrillogenesis and improved prognosis.

## Material and Methods

### Ethics statement and Animals

All animal experiments were carried out according to the University of Alberta Animal Policy and Welfare Committee and the Canadian Council on Animal Care Guidelines. The approval for use of animals in research was granted by the Animal Care and Use Committee for Health Sciences, a University of Alberta ethics review committee. The protocol was approved by the Committee (Permit AUP297). Animals were monitored daily for responsiveness, body conditions, respiration, physical appearance and mobility. Animals were euthanized when they met specific criteria or showed signs of distress. No animal died prior to experimental endpoints. Total of 162 animals were used in the study (equal number of male and female mice). All animal experimentation was carried out working closely with University of Alberta animal facility staff and veterinarian. The details of transgenic mice carrying a transgene that directs calreticulin overexpression in the heart was previously described [26]. To induce cardiac overexpression of calreticulin, transgenic mice were fed tamoxifen for three weeks [26]. These animals are designated as Heart^CRT+^ mice. Some animals received in their diet tauroursodeoxycholic acid (TUDCA; TCI America, T1567) dissolved in water at 2 mg/ml. Fresh TUDCA solution was administered every other day for three weeks as indicated in the text and the Figures.

### Microarray analysis

Total RNA was isolated from transgenic and control heart homogenates using TRIzol reagent (Invitrogen) according to the manufacturer’s instructions and as described previously [26]. A double strand cDNA library was constructed from 300 ng of purified RNA using a library synthesis kit (Sigma) with the following incubations, 18°C for 10 min, 25°C for 10 min, 37°C for 30 min, 42°C for 10 min, and 70°C for 20 min. After adding the PCR amplification mixture to the sample, the PCR reaction cycle was performed as follows, denaturation at 94°C for 2 min, and 17 cycles of extension at 94°C for 30 sec, and 70°C for 5 min, and the product was cleaned up using the GenElute PCR Clean up kit (Sigma), and the Nanoassay was examined using ND-1000 software (NanoDrop Technologies) to verify the quality of synthesized cDNA. For probe labeling, purified cDNA was mixed with Cy3 primer and dNTP/Klenow fragment (50 U/ul) (NimbleGen one color labelling kit), and incubated for 2 h at 37°C with a heated lid. Hybridization to mouse MM9 Expression Array (12x135k, NimbleGen) was carried out at 42°C for 20 h in an HX12 Mixer (Roche), to acquire uniform signal and high sensitivity with minimal sample volume across the array surface. After washing, arrays were scanned using NimbleGen MS 200 software, and the data were extracted using NimbleScan software. Array normalization was performed using the quantile normalization method of Bolstad et al. [27]. Normalized expression values for the individual probes were used to obtain the expression values for a given open reading frame (ORF) by using the robust multiarray average (RMA) procedure [28]. *n*-fold change ratios for a particular gene in calreticulin transgenic and control heart were calculated using the RMA-processed expression values (RMA calls). Finally, the data were clustered and analyzed through the use of DAVID [29] and IPA (Ingenuity® Systems) software. Microarray data are available at the NCIB accession number GSE82188.

### Real-time RT-PCR and Immnoblot analysis

A Rotor-Gene RG-3000 or Rotor-Gene Q (Corbett Research) and SYBR Green Supermix (Quanta BioSciences) were used for real-time RT-PCR experiments. The final quantitation of the amount of target (Ct value) in a real-time RT-PCR reaction was converted to the amount of transcript and normalized by glyceraldehyde 3-phosphate dehydrogenase (GAPDH). PCR primers used in this study are listed in S2 Table. For Immnoblot analysis, proteins from control and Heart^CRT+^ hearts were homogenized, separated by SDS-PAGE, and followed by immunoblotting. Immunoreactive protein bands were detected using peroxidase-conjugated secondary antibodies followed by a standard enhanced chemiluminescence reaction [26]. Blots were probed with the following antibodies: goat anti-calreticulin; mouse anti-human influenza hemagglutinin (1:500, Santa Cruz Biotechnology); rabbit anti-collagen type I and III (1:500, Chemicon), rabbit anti-Smad2 and 3 (1:1000, Cell Signaling), rabbit anti-phospho-Smad2 (Ser^465/467^) (1:1000, Cell Signaling), rabbit anti-phospho-Smad3 (Ser^423/425^) (1:1000, Cell Signaling), rabbit anti-TGFβ1 (1:1000, Abcam), rabbit anti-periostin (1:1000, Abcam), rabbit anti-fibronectin (1:1000, Abcam); rabbit anti-BiP (1:1000, Abcam); mouse anti-ATF6 p50 (1:500, Novus Biologicals); mouse anti-phospho-eIF2α (1:500, Santa Cruz Biotechnology); mouse anti-CHOP (1:500, Santa Cruz Biotechnology) antibodies; and rabbit anti-GAPDH antibodies (1:1000, Abcam).

### Trichrome staining and immunohistochemistry

Cardiac tissue was fixed in 10% formalin in phosphate buffered saline. Paraffin-embedded tissues were sectioned by 5 μm and mounted on the slide. Fibrosis was analyzed with revised Gomori’s trichrome staining [30]. Sectioned hearts were placed in filtered Bouin’s solution at 60°C for 30 min and let sit for another 30 min at room temperature. After washing the slide with water, the slide was stained with filtered trichrome for 20 min, and placed in 0.5% acetic water for 2 min. Slides were observed by light microscopy (Axioskop 2, Zeiss), and images were captured using QCapture Pro 5.0 (QImaging), and fibrotic areas were analyzed using ImageJ software. For immunohistochemistry, paraffin-embedded slides were deparaffinised and rehydrated prior to antigen retrieval. To break methylene bridges formed during fixation, heat-induced epitope retrieval was carried out in a buffer containing 10 mM Tris, pH 9.0, 1 mM EDTA and 0.05% Tween 20. The tissue sections were then incubated overnight with primary antibodies [1:40 and 1:100 dilutions in TBS with 1% BSA for rabbit anti-collagen type I (Millipore) and rabbit anti-periostin (Abcam), respectively] in a humidified chamber. Antigen-antibody complexes were visualized with incubation of Fluorescein isothiocyanate-conjugated secondary antibodies (goat anti-rabbit, Abcam) for 1 h at room temperature. Stained images were captured on a Leica TCS SP5 confocal microscope. Nuclei were visualized with DAPI.

### Statistical analysis

All data are presented as mean ± standard error of the mean. Statistical analysis was performed using SigmaPlot 10.0 (Systat Software) with the paired t-test and One-way analysis of variance. Statistical significance was accepted at a p<0.05 value.

## Results

### Heart^CRT+^ hearts develop cardiac fibrosis

**Fig 1:**
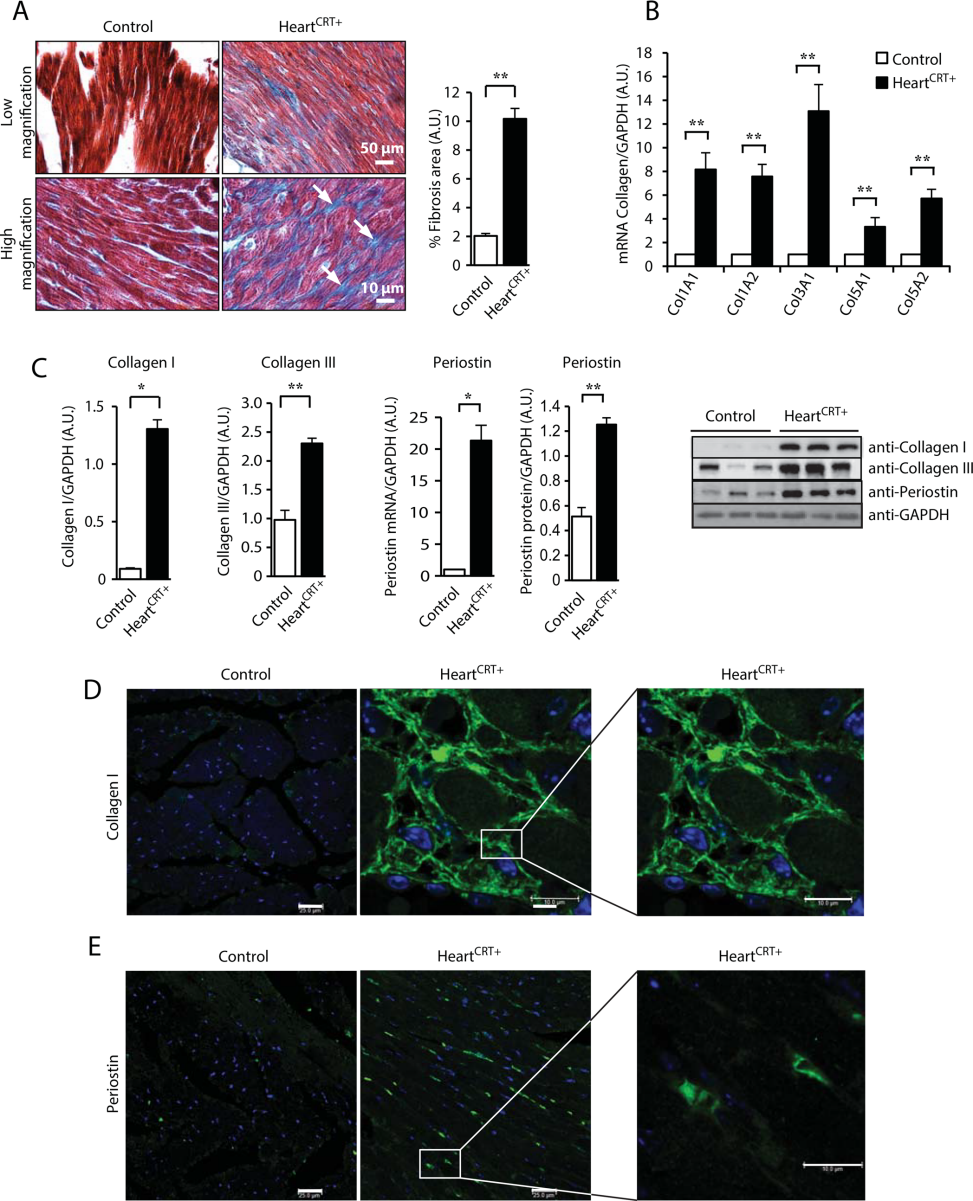
Fibrosis and increase abundance of collagen and periostin in heart with increased expression of calreticulin. **(A)** Gomori’s trichrome staining for collagen depositions in control and Heart^CRT+^ myocardium. The arrows indicate the location of the blue staining for collagen. Quantitative analysis of the percentage of areas with collagen deposition in control and Heart^CRT+^ hearts is shown to the right. **p<0.01. Data are representative of 6 biological replicates. **(B)** Abundance of fibrillar collagen mRNA was analyzed by Q-PCR. Values were normalized to glyceraldehyde 3-phosphate dehydrogenase (GAPDH) mRNA. Col1A1, collagen type I, alpha 1; Col1A2, collagen type I, α2; Col3A1, collagen type III, α1; Col5A1, collagen type V, α1; Col5A2, collagen type V, α2. ** p<0.01. Data are representative of 9 biological replicates. **(C)** Immunoblot analysis and quantification of the abundance of collagen type I in control and Heart^CRT+^ hearts. Anti-GAPDH were used as a loading control. **p<0.01. Data are representative of 6 biological replicates. (**D)** Abundance of collagen type III in control and Heart^CRT+^ hearts. Anti-GAPDH antibodies were used as a loading control. *P<0.05. Data are representative of 6 biological replicates. (**E**) Subcellular distribution of type I collagen by immunohistochemistry in cardiac tissue cross-sections from control and Heart^CRT+^ hearts. Green color represents staining for type I collagen. **(F)** Abundance of periostin mRNA in control and Heart^CRT+^ hearts. **p<0.01. Data are representative of 9 biological replicates. Immnoblot and quantitative analyses of periostin in Heart^CRT+^ and control transgenic hearts. *p<0.05. Anti-GAPDH antibodies were used as a loading control. Data are representative of 6 biological replicates. (**G)** Cellular distribution of periostin in cardiac tissue cross-sections from control and Heart^CRT+^ hearts. Nuclei were visualized with DAPI staining. 3–5 animals were used for each analysis.

In this study we created a mouse model with forced cardiomyocytes overexpression of calreticulin (designated throughout this manuscript as Heart^CRT+^) that have disrupted ER homeostasis in the heart and develop dilated cardiomyopathy and heart failure [26]. A global gene-expression profile microarray analysis (44,170 target genes) of the Heart^CRT+^ hearts revealed an increase in the activation of genes not only associated with cardiac dilation but also with cardiac fibrosis (S1 Fig and S1 Table). Indeed, trichrome staining of the interstitial myocardium and quantitative analysis of the fibrotic areas showed a large deposition of collagen in Heart^CRT+^ hearts (Fig 1A), thus confirming that increased abundance of calreticulin in the heart stimulates cardiac fibrosis. There was also a large increase in the abundance of collagen encoding transcripts Col1A1, Col1A2, Col3A1, Col5A1 and Col5A2 (Fig 1B). Linear regression analysis demonstrated strong concordance between the results obtained by Q-PCR (Fig 1B) and microarray analysis (S1 Fig), with an R^2^ = 0.845 (S2 Fig). Immunoblot analysis showed increased abundance of collagen I and collagen III proteins (Fig 1C and 1D), which are major components of the myocardial collagen network [31]. Immunofluorescence imaging showed robust staining for type-I collagen in Heart^CRT+^ heart sections (Fig 1E). We also found a large increase in the abundance of periostin protein and mRNA in Heart^CRT+^ hearts (Fig 1F), a secreted ECM protein involved in muscle fibrosis [32]. Increased staining of periostin was seen in cardiac muscle sections in Heart^CRT+^ mice (Fig 1G). Other components of ECM were also increased in abundance in the Heart^CRT+^ hearts (S3 Fig). Fibronectin protein (S3A Fig) and mRNA (S3B Fig), along with mRNA for fibrillin, elastin, tissue inhibitors of metalloproteinase (TIMP-1) and MMP2 (but not MMP9) (S3B, S3C and S3D Fig) were all increased in abundance in the Heart^CRT+^ hearts. These results indicate that Heart^CRT+^ developed severe fibrosis.

### Activation of TGFβ1 is the cause of cardiac fibrosis in Heart^CRT+^

**Fig 2:**
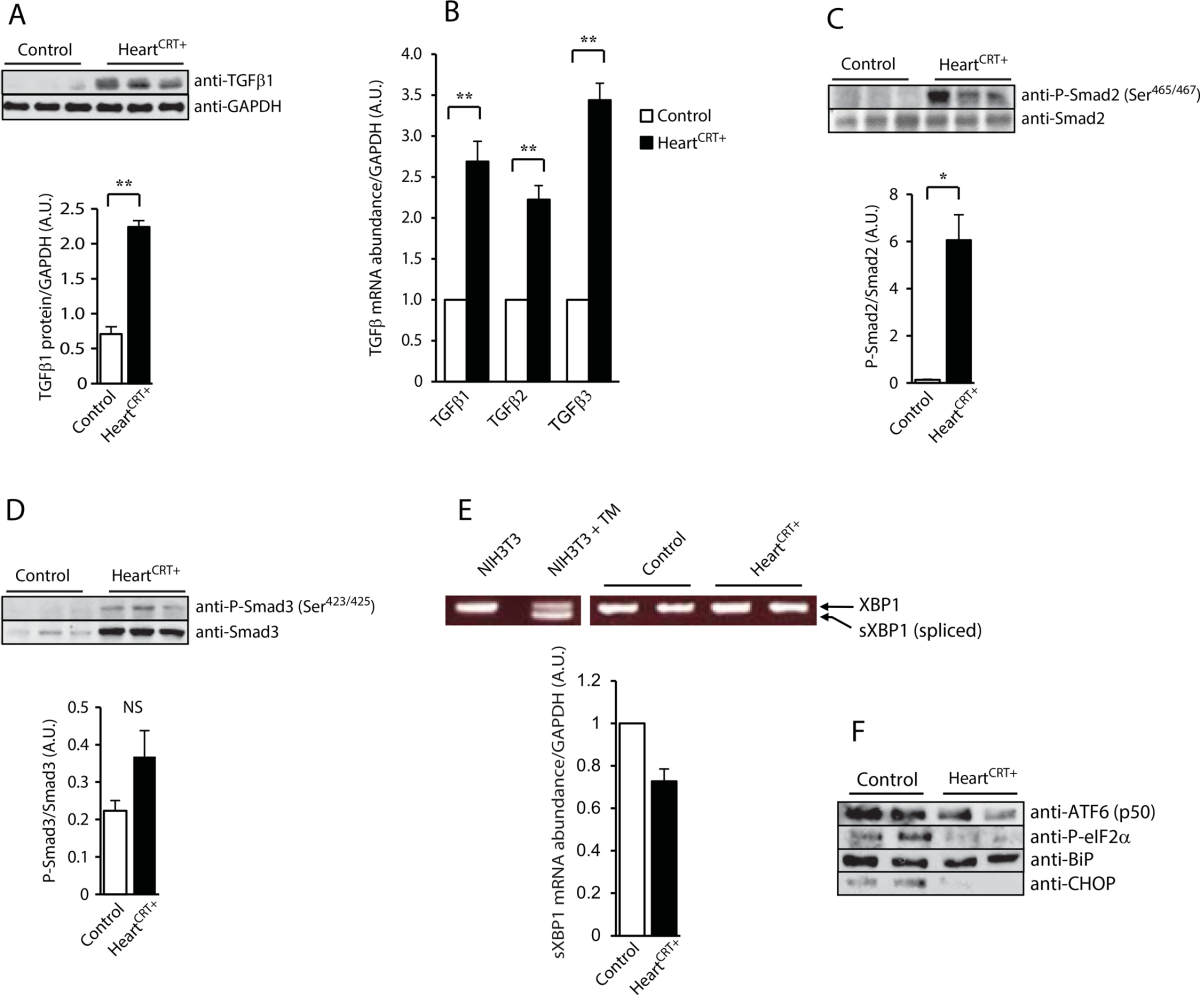
TGFβ1 and unfolded protein response (UPR) in calreticulin expressing hearts. (**A)** Immnoblot analysis and quantification of TGF-β1 protein in control and Heart^CRT+^ hearts. Antibodies to glyceraldehyde 3-phosphate dehydrogenase (GAPDH) were used as a loading control. **p<0.01. Data are representative of 6 biological replicates. (**B)** Real-time Q-PCR analysis for TGF-β1, β2 and β3 transcripts. **p<0.01. Data are representative of 6 biological replicates. (**C)** Immunoblot analysis of Smad2 and phospho-Smad2 (P-Smad2) at Ser^465^ and Ser^467^ antibodies from control and Heart^CRT+^ hearts. Quantitative analysis of P-Smad2/Smad2 expression ratio shown in. Anti-GAPDH antibodies were used as a loading control. * p<0.05. Data are representative of 6 biological replicates. (**D)** Immnuoblot analysis of Smad3 and phospho-Smad3 (P-Smad3) at Ser^423^ and Ser^425^ antibodies from control and Heart^CRT+^ hearts. Quantitative analysis of P-Smad3/Smad3 ratio. Anti-GAPDH antibodies were used as a loading control. NS, not significant. Data are representative of 6 biological replicates. **(E**) Real-time Q-PCR analysis of spliced XBP1 (sXBP1) mRNA abundance. The abundance of the sXBP1 transcripts in Heart^CRT+^ was slightly decreased (0.73±0.06 vs. control). **p<0.01. Data are representative of 6 biological replicates. NIH3T3 cells treated with tunicamycin (TM), an activator of unfolded protein response, were used as a positive control for the XBP1 splicing analysis. **(F**) Immnunoblot analyses of UPR markers were carried out with specific anti-ATF6 p50 fragment, anti-P-elF2α, anti-BiP and anti-CHOP antibodies. Anti-GAPDH antibodies were used as a loading control. Data are representative of 6 biological replicates.

TGFβ1, a pleiotropic cytokine associated with cardiac fibrosis [33], was identified as a high scoring regulatory molecule in the Ingenuity Pathway Analysis of the Heart^CRT+^ heart transcriptome network (S1 Table). Immunoblot and Q-PCR analysis of tissue and mRNA isolated from Heart^CRT+^ hearts further confirmed microarray data showing increased abundance of TGFβ1 protein and mRNA (Fig 2A and 2B) as well as increased mRNA abundance of TGFβ2 and TGFβ3 isoforms (Fig 2B). Receptor-regulated Smads (R-SMAD, Smad2/3) are well-known downstream effectors of the TGFβ1 signaling pathway in cardiac fibrosis [34, 35]. The degree of phosphorylation of Smad2 at serine^465^ and serine^467^ (Ser^465/467^) and Smad3 at serine^423^ and serine^425^ (Ser^423/425^) was substantially increased in Heart^CRT+^ hearts (Fig 2C and 2D). The abundance of the phospho-Smad2 Ser^465/467^ was especially high (>47-fold increase). The increased abundance of TGFβ1 and accompanying activation of Smads in Heart^CRT+^ hearts indicate that the canonical TGFβ/Smad signaling pathway was activated.

### UPR pathway is transiently activated in response to calreticulin overexpression

**Fig 3:**
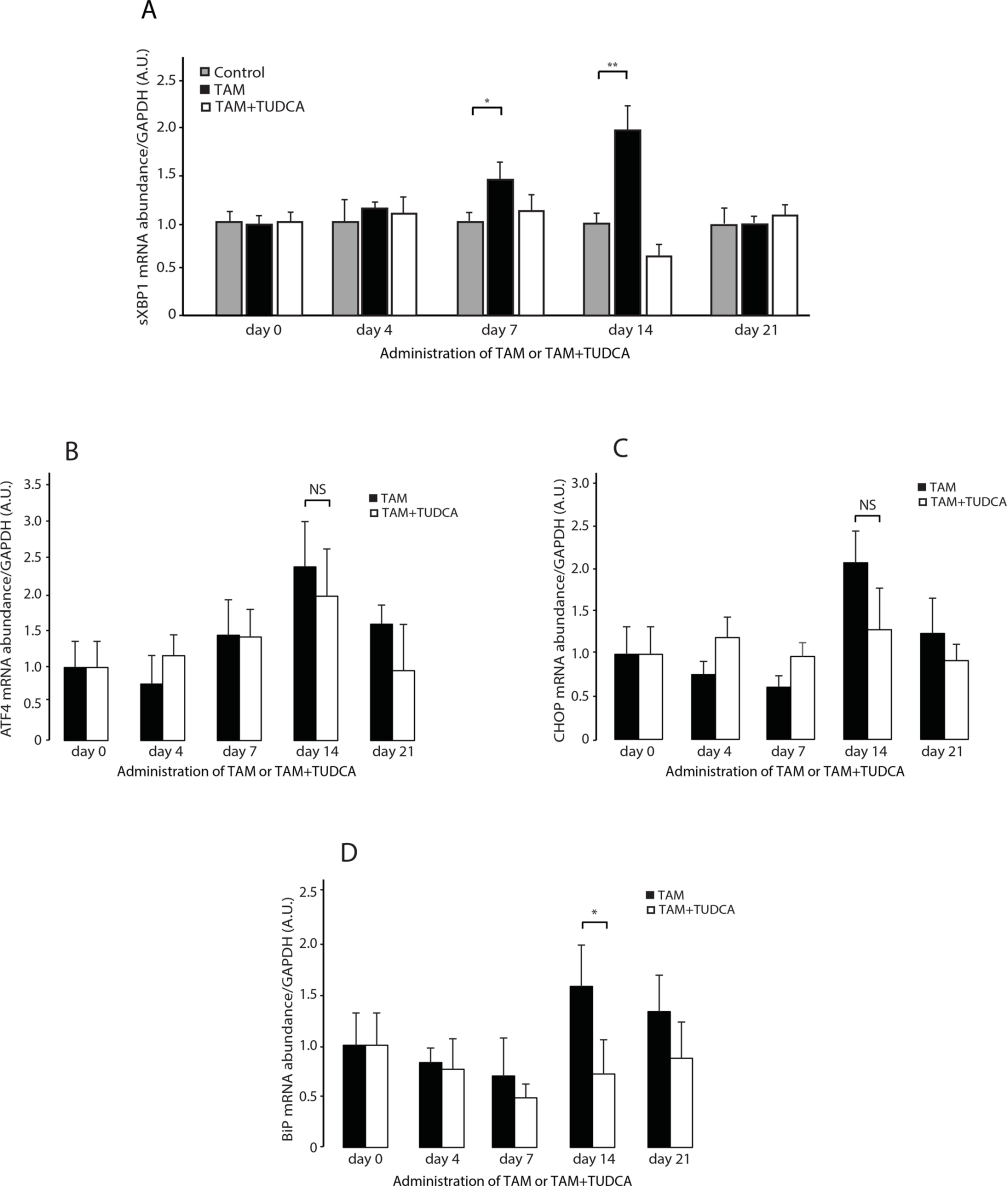
XBP1 splicing and abundance of ATF4, CHOP and BiP mRNA in the Heart^CRT+^ myocardium. **(A)** Real-time Q-PCR analysis of XBP1 splicing as a function of treatment time. Mice were fed tamoxifen (TAM) to induced overexpression of calreticulin in the hearts of transgenic calreticulin mice. Immnunoblot probed with anti-calreticulin antibodies shows the abundance of the calreticulin protein (CRT, arrow) in transgenic calreticulin hearts. *, non-specific immunoreactive protein band. The graph below shows the abundance of spliced XBP1 (sXBP1) in the hearts of control animals (gray bars), and the TAM fed animals (black bars) and animals fed TAM plus tauroursodeoxycholic acid (TUDCA) (white bars). Day 7 *p = 0.0233, Day 14 **p = 0.0028. Data are representative of 6 biological replicates. 3–5 animals were used for each analysis. **(B,C,D)** Real-time Q-PCR analysis of abundance of ATF4 **(B)**, CHOP **(C)** and BiP **(D)** mRNA as a function of treatment time. Mice were fed tamoxifen (TAM) to induced expression of calreticulin in the hearts of transgenic calreticulin mice. TUDCA, tauroursodeoxycholic. For day14 BiP mRNA graph *p = 0.0467, NS, not significant.

Calreticulin is an ER resident Ca^2+^ buffer and molecular chaperone [36]. Increased calreticulin abundance in the heart is expected to alter ER proteostasis, Ca^2+^ homeostasis and consequently induce the UPR pathway, an ER stress coping response [2]. Yet surprisingly, after 21 days of induction of calreticulin expression, there was no detectable activation of any of the UPR signaling arms (IRE1α PERK or ATF6) in Heart^CRT+^ hearts (Fig 2E and 2F). In fact, UPR-induced processed ATF6 was actually decreased (Fig 2F) and phosphorylated eIF2α, a marker of PERK activity, was reduced in Heart^CRT+^ hearts (Fig 2F). Abundance of BiP, a protein that is increased by the UPR, was not affected (Fig 2F). CHOP levels were at the limit of detection in Heart^CRT+^ hearts (Fig 2F). Thus, contrary to expectations, no activation of the classical UPR arms in Heart^CRT+^ hearts was evident at 21 days after initiation of calreticulin overexpression. Therefore, we checked whether there was transient activation of UPR in Heart^CRT+^ hearts at earlier time points. Specifically, we monitored the activation of IRE1α, as a function of temporal induction of calreticulin abundance in hearts. After 7 days, IRE1α-dependent XBP1 splicing increased concomitant with the rise in calreticulin abundance in transgenic hearts (Fig 3A). The abundance of spliced XBP1 was the highest at day 14 (Fig 3A) and subsequently returned to the initial level by day 21 (Fig 2E and Fig 3A). Abundance of ATF4 and CHOP mRNA was also increased at day 14 but in contrast to XBP1 splicing it was not sensitive to TUDCA administration (Fig 3B, 3C and 3D). The level of BiP mRNA, as expected, was increased at day 14 (Fig 3B, 3C and 3D) and the abundance of BiP mRNA was reduced at day 14 in animals treated with TUDCA (Fig 3B, 3C and 3D). These results illustrate that induction of calreticulin abundance in murine hearts was linked to a transient increase in UPR signaling. Importantly, only XBP1 splicing, a substrate for IRE1α (Fig 3A) and the abundance of BiP mRNA were sensitive to administration of TUDCAin the Heart^CRT+^ hearts.

### Pharmacological inhibition of IRE1αactivity ameliorates cardiac fibrosis in Heart^CRT+^

**Fig 4:**
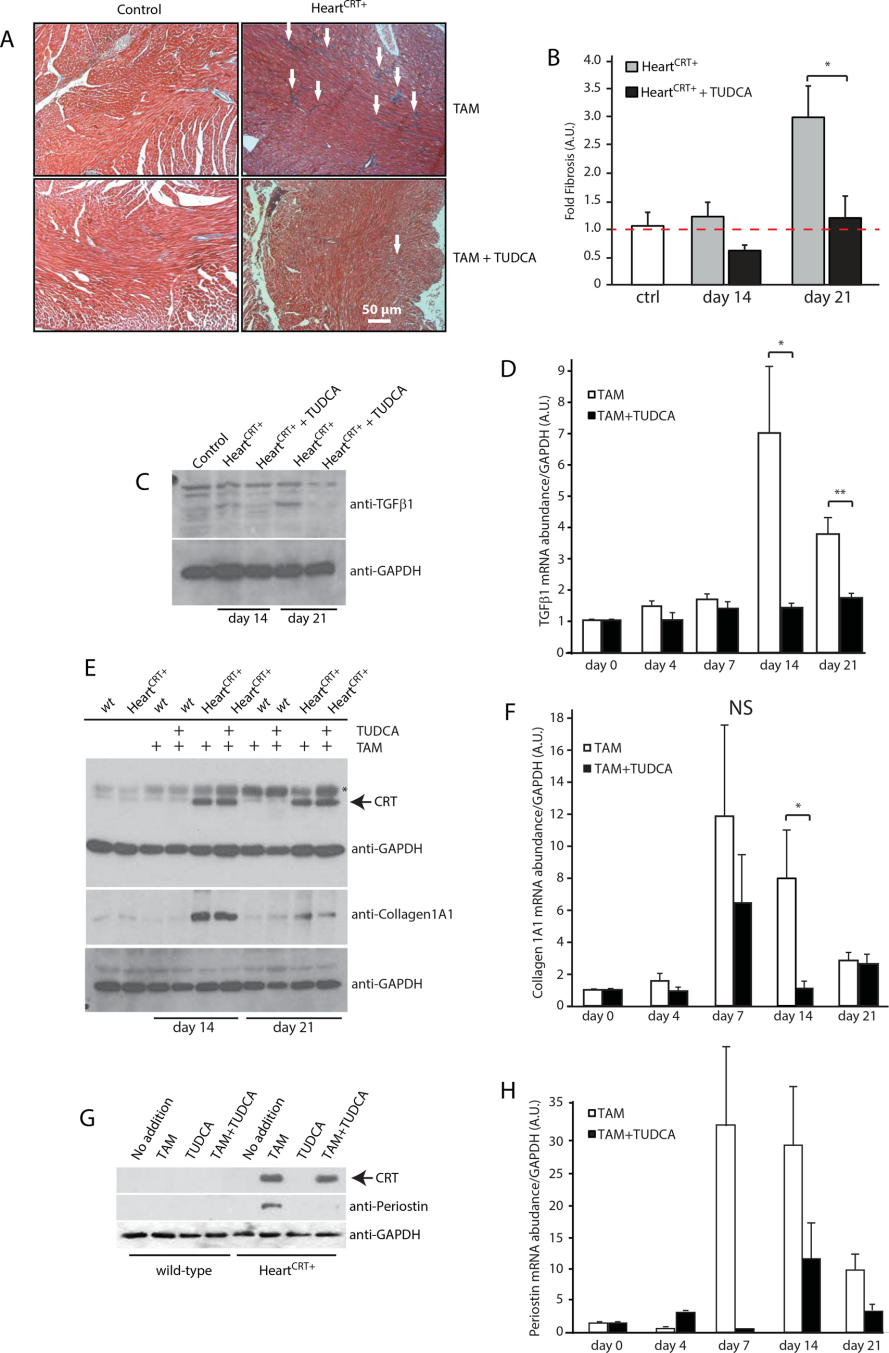
Tauroursodeoxycholic acid (TUDCA) prevents cardiac fibrosis in Heart^CRT+^ mice. **(A)** Trichrome staining for collagen deposition in the myocardium of control, transgenic and Heart^CRT+^ mice fed TUDCA. The arrows indicate the location of blue staining for collagen. (**B)** Quantitative analysis of the percentage of area with collagen deposition in control (ctrl), and Heart^CRT+^ animals fed tamoxifen (TAM) or TAM+ TUDCA at day 14 and day 21 *p<0.01. Data are representative of 6 biological replicates. **(C)** Immnunoblot analysis and quantification of TGFβ1 protein in hearts from Heart^CRT+^ mice fed TAM or TAM+TUDCA. Antibodies to glyceraldehyde 3-phosphate dehydrogenase (GAPDH) were used as a loading control. Data are representative of 6 biological replicates. (**D)** Real-time Q-PCR analysis for TGFβ1 transcript in hearts from Heart^CRT+^ animals fed TAM or TAM+ TUDCA. * p = 0.0254 ** p = 0.0148. Data are representative of 6 biological replicates. TAM samples were normalized to Day 0 and TAM+TUDCA samples were normalized to the corresponding day in TAM treatment to highlight the TUDCA effect. (**E)** Abundance of collagen type 1A1 in wild-type (*wt*) and transgenic mice fed TAM+TUDCA for 14 or 21 days. Antibodies to human influenza hemagglutinin (HA) were used to probe for the abundance of HA-tagged recombinant calreticulin (CRT). Anti-GAPDH antibodies were used as a loading control. (**F)** Abundance of collagen 1A1 mRNA in hearts from Heart^CRT+^ animals fed TAM or TAM+TUDCA. NS, not significant. *p = 0.0424. Data are representative of 6 biological replicates. TAM samples were normalized to Day 0 and TAM+TUDCA samples were normalized to corresponding day in TAM treatment to highlight the TUDCA effect. (**G)** Western blot analysis of periostin in hearts from TAM+TUDCA fed mice. Anti-HA antibodies were used to probe for abundance of HA-tagged recombinant calreticulin (CRT). Anti-GAPDH antibodies were used as a loading control. (**H)** Abundance of periostin mRNA in hearts from Heart^CRT+^ animals fed TAM or TAM+TUDCA. Data are representative of 6 biological replicates. TAM samples were normalized to Day 0 and TAM+TUDCA samples were normalized to corresponding day in TAM treatment to highlight the TUDCA effect. 3–5 animals were used for each analysis.

We next asked if inhibiting the early activation of the IRE1α in Heart^CRT+^ hearts could improve the cardiac pathology outcome. Calreticulin overexpression in the heart was induced in the presence of TUDCA, the taurine-conjugated form of ursodeoxycholic acid [37], known to inhibit the UPR [38–41]. TUDCA treatment reduced XBP1 splicing and efficiently prevented the activation of IRE1α in Heart^CRT+^ hearts (Fig 3A, white bars). In parallel, trichrome staining of the interstitial myocardium (Fig 4A) and quantitative analyses of the fibrotic areas (Fig 4B) revealed a striking reduction in the collagen deposition in hearts of Heart^CRT+^ mice treated with TUDCA, concomitant with the large reduction of protein and mRNA abundance for collagen 1A1 (Fig 4E and 4F) and periostin (Fig 4G and 4H). Abundance of TGFβ1 protein (Fig 4C) and mRNA (Fig 4D), the key regulator of the fibrotic response in the heart, was also dramatically reduced in TUDCA treated Heart^CRT+^ mice. Moreover, Heart^CRT+^ mice treated with TUDCA displayed remarkable improvement in behaviour and rate of physical activity compared to the listless behaviour of non-TUDCA-treated Heart^CRT+^ mice (S1 Video).

## Discussion

This study demonstrates for the first time that inhibition of the unfolded protein response using the pharmacological agent TUDCA decreased cardiac fibrosis in a mouse model of heart failure induced by calreticulin overexpression. Myocardial fibrosis is a hallmark of cardiomyopathy and contributes to cardiac cell death, ventricular arrhythmias, left ventricle dysfunction and heart failure. The role of ER stress and components of the ER protein quality control in cardiac pathology is only just emerging [2]. We recently proposed that activation of ER stress coping responses, including UPR, leading to the successful recovery of homeostasis at the cellular level could lead to pathological outcomes at the organismal level [2]. This study supports this notion, showing that transient activation of IRE1α in the heart led to permanent modification of the cellular program in the organ, culminating in cardiac fibrosis. Although activation of UPR may have promoted cardiomyocyte survival, the permanent activation of TGFβ1 pathways led to cardiac pathology and heart failure. The silencing IRE1α activity by TUDCA specifically prevented the activation of the TGFβ1 pathways *in vivo* thereby preventing the ensuing fibrillogenesis.

Increased abundance of calreticulin results in increased Ca^2+^ buffering capacity of the ER, higher concentration of the ER luminal free Ca^2+^, delayed store-operated Ca^2+^ entry and reduced Ca^2+^ movement to mitochondria with little impact on protein folding [42–45], all hallmarks of impaired ER and cellular Ca^2+^ homeostasis. The link between ER homeostasis and the molecular mechanisms underlying cardiac remodelling due to deposition of ECM proteins leading to heart failure is not known. In our study, whereby ER Ca^2+^ was increased intentionally by calreticulin overexpression, disruption of ER homeostasis was seen to cause the transient activation of UPR coping response ultimately manifested as cardiac fibrosis at the organ level. Our findings offer insights into the pathophysiology of cardiac fibrosis, and suggest that a key stimulus for myocardial fibrosis is disrupted by ER Ca^2+^ homeostasis. In full support of our *in vivo* observations calreticulin-deficient mouse embryonic fibroblasts, which have reduced ER Ca^2+^ content, showed concomitant reduction in transcript levels for fibrillar collagen I and III, and less soluble collagen deposition [46]. In turn fibroblasts with increased abundance of calreticulin, which have increased ER Ca^2+^ concentration, have increased collagen type I transcript and protein [46]. In a renal fibrosis rat model, calreticulin abundance is also increased in tubular epithelial cells before ECM deposition and it may promote a profibrotic cellular phenotype [47, 48]. Activation of UPR may also play a role in fibrotic diseases of kidney, liver, lung but the direct link to the TGFβ1 pathway needs to be established. [49, 50]. Furthermore, a recent study indicates that hepatitis C virus infection induces TGFβ1 expression through the UPR pathway and this may play a role in liver fibrosis [51].

Cardiac fibrosis is presently difficult to treat in the clinic, and is a common final pathway for many cardiac diseases leading to heart failure. Management of cardiac fibrosis is challenging due to the induction of multiple pathways by neurohormonal and cytokine factors which contribute to cardiac fibrillogenesis in cardiomyopathy [52–54]. Proteins such as TGFβ1, endothelin-1, angiotensin II, connective tissue growth factor and platelet-derived growth factor (PDGF) may be the key contributors to cardiac fibrosis [52–54]. These proteins collectively activate mesenchymal cells (fibroblasts) causing increased production and deposition of ECM components [52, 53]. Previous attempts to identify drugs that are effective therapies against cardiac fibrosis have been unsuccessful [54]. We here now show that cardiac fibrosis is a preventable process in the failing heart, and this is achievable by early pharmacologic inhibition of the IRE1α pathway of UPR.

## Supporting Information

S1 Fig Microarray analysis of Heart^CRT+^ hearts. **A.** Functional annotation chart by biological process in calreticulin transgenic hearts analyzed by DAVID (The Database for Annotation, Visualization and Integrated Discovery, Bioinformatics Resources 6.7). **B.** Cardiotoxicity analysis of microarray data by the IPA^®^ software.
(EPS)

S2 Fig Correlation of expression ratios between microarray and real-time RT-PCR experiments. The calculated expression ratios (*n*-fold changes) are shown for microarray experiments (horizontal axis) and real-time Q-PCR (vertical axis). The best-fit linear regression curve is shown along with the coefficient of determination, R^2^ = 0.845. [R^2^ = 1-SSerr/SStot (SSerr: the sum of squares of residuals and SStot: the total sum of squares)].
(EPS)

S3 Fig The abundance of extracellular matrix components in the Heart^CRT+^ myocardium. **A.** Immunoblot analysis and quantification of the abundance of fibronectin in control and Heart^CRT+^ hearts. GAPDH, glyceraldehyde 3-phosphate dehydrogenase. **p<0.01. Data are representative of 6 biological replicates. **B.** Q-PCR analysis of fibronectin1, fibrillin1 and elastin transcripts. *p<0.05, **p<0.01. All data are representative of 6 biological replicates. **C** and **D**. Q-PCR analysis of tissue inhibitors of metalloproteinase (TIMP1) (**C**), and metalloproteinases MMP2 and MMP9 (**D**). **p<0.01. All data are representative of 6 biological replicates.
(EPS)

S1 Table Selected high scored regulatory molecules in the Heart^CRT+^ hearts. (DOCX)

S2 Table Nucleotide sequence of DNA primers used in this study for real-time RT-PCR analysis. (DOCX)

S1 Video Physical activity of Heart^CRT+^ animals fed TUDCA. Heart^CRT+^ mice receiving TUDCA displayed significant improvement in behaviour and rate of physical activity. Two animals are shown in the video, both the same litter are shown, only one of the revised TUDCA in the diet (Heart^CRT+^ + TUDCA).
(MP4)
