# Supplementary material for: Inhibition of the Unfolded Protein Response Mechanism Prevents Cardiac Fibrosis
Source: PLoS One. 2016 Jul 21;11(7):e0159682. doi: 10.1371/journal.pone.0159682 (PMC4956237; doi:10.1371/journal.pone.0159682)
Supplement: S1 Table — (DOCX) [file pone.0159682.s004.docx]

**S1Table**. Selected high scored regulatory molecules in the Heart^CRT+^ hearts

| **ID** | **Gene Name** |
| --- | --- |
| AK159377 | AE binding protein 1 |
| AJ250684 | CD22 antigen; hypothetical protein LOC100047973 |
| BC075622 | CD24a antigen |
| AK170937 | CD33 antigen |
| BC006607 | CD34 antigen |
| BC051388 | CD44 antigen |
| BC041794 | FAT tumor suppressor homolog 1 (Drosophila) |
| BC016549 | Ly6/Plaur domain containing 3 |
| BC021772 | PERP, TP53 apoptosis effector |
| BC032877 | WNT1 inducible signaling pathway protein 2 |
| BC057043 | anthrax toxin receptor 1 |
| BC022107 | cadherin 2; similar to N-cadherin |
| BC128368 | cartilage oligomeric matrix protein |
| AK142745, BC112409 | catenin (cadherin associated protein), alpha 3 |
| BC006650 | chemokine (C-X3-C motif) ligand 1 |
| BC156500 | collagen, type VI, alpha 1 |
| AK132540, BC034414 | collagen, type VI, alpha 2 |
| BC011061 | collagen, type VIII, alpha 1 |
| AK028536, AK076278 | collagen, type XII, alpha 1 |
| AY221110, AK052963 | collagen, type XIV, alpha 1 |
| BC156474 | collagen, type XV, alpha 1 |
| AK138698 | discoidin domain receptor family, member 1 |
| BC005481 | elastin microfibril interfacer 1 |
| AK147315, BC099373 | fibronectin 1 |
| BC006636 | fibulin 5 |
| BC095941 | fibulin 7 |
| BC026375 | glycoprotein (transmembrane) nmb |
| BC058716 | integrin alpha 11 |
| AK139690 | integrin alpha 9 |
| BC058246, AK087476 | integrin beta 5 |
| AK019511, AF115376, AK008867, AK036439 | integrin beta 6 |
| BC020152 | integrin, beta-like 1 |
| AK155769 | laminin, beta 3 |
| BC026985, AB035509 | melanoma cell adhesion molecule |
| AK075659, BC022666 | microfibrillar-associated protein 4 |
| X14526 | neural cell adhesion molecule 1 |
| BC053713, NM_001111324, AF009366 | neural precursor cell expressed, developmentally down-regulated gene 9 |
| BC054746 | nidogen 2 |
| BC095997 | osteomodulin |
| BC026772 | paraspeckle protein 1 |
| BC031449, AY651928 | periostin, osteoblast specific factor |
| M83997, AK147396 | perlecan (heparan sulfate proteoglycan 2) |
| BC026551 | podoplanin |
| BC095964, XM_001478164 | predicted gene 3655; B-cell leukemia/lymphoma 2 |
| AY861419, AY861420 | protocadherin 7 |
| AK147451 | protocadherin 9 |
| BC127609 | protocadherin beta 12 |
| BC059821 | protocadherin beta 14 |
| AK165965 | secreted phosphoprotein 1 |
| BC021155 | similar to TNF-stimulated gene 6 protein; tumor necrosis factor alpha induced protein 6 |
| BC050917, BC042422, M87276 | thrombospondin 1; similar to thrombospondin 1 |
| BC053023 | thrombospondin 3 |
| BC011055, BC011170 | transforming growth factor, beta 2 |
| BC053018 | trophinin |
| BC025860 | tumor necrosis factor receptor superfamily, member 12a |
| BC046310 | tweety homolog 1 (Drosophila) |
| AK014525, AK034871 | versican |
