# Supplementary material for: Inhibition of the Unfolded Protein Response Mechanism Prevents Cardiac Fibrosis
Source: PLoS One. 2016 Jul 21;11(7):e0159682. doi: 10.1371/journal.pone.0159682 (PMC4956237; doi:10.1371/journal.pone.0159682)
Supplement: S2 Table — (DOCX) [file pone.0159682.s005.docx]

**S2 Table.** Nucleotide sequence of DNA primers used in this study for real-time RT-PCR analysis

| Gene | Forward primers | Reverse primers |
| --- | --- | --- |
| Calreticulin | 5’-TTACGCACTGTCCGCCAAA-3’ | 5’-GCTCATGCTTCACCGTGAACT-3’ |
| Col1A1 | 5’-TGTCCCAACCCCCAAAGAC-3’ | 5’-CCCTCGACTCCTACATCTTCTGA-3’ |
| Col1A2 | 5’-CCAGCGAAGAACTCATACAGC-3’ | 5’-GGACACCCCTTCTACGTTGT-3’ |
| Col3A1 | 5’-AAGTTCACCAGCAACAGCAG-3’ | 5’-TTGGTTAGCCATGTAGAGCG-3’ |
| Col5A1 | 5’-TAAGGAGATGCCCTCTACCGAG-3’ | 5’-GTGCCTTTCCGATGGATTAAAG-3’ |
| Col5A2 | 5’-TACAAAGCTGATGCAGGACC-3’ | 5’-TATTGGGCCAGTTTGTTTCA-3’ |
| TGFβ1 | 5’-CACCTGCAAGACCATCGACAT-3’ | 5’-GAGCCTTAGTTTGGACAGGATCTG-3’ |
| TGFβ2 | 5’-AATGACAACGATGACGACCA-3’ | 5’-GATGCAGACTAACGCCTTCC-3’ |
| TGFβ3  MMP-2  MMP-9 | 5’-TAGCGAGTGGACATTGTGAGTGGC-3’  5’-TAGTGATGGTTCCCCTCCTC-3’  5’-CGTCTGAGAATTGAATCAGC-3 | 5’-GGGGACTTTGGCTTGGTAAACTG-3’  5’-TACTTGTTTGCCATTTCCCA-3’  5’-AGTAGGGGCAACTGAATACC-3’ |
| Periostin  Fibronectin  Fibrillin  Elastin | 5’-AACCCACATTGCATGAGAAA-3’  5’-AGCAGTGGGAACGGACCTAC-3’  5’-GGACACGATGCGCTGAAAGG-3’  5’-CAAGTCGGAGCTGGCATCGG-3’ | 5’-CAGGAAGAGGCTGGAAAAAC-3’  5’-ACGTAGGACGTCCCAGCAGC-3’  5’-CAGGAATGCCGGCAAATGGG-3’  5’-GTGGGAACTCCAGGGAGCAC-3’ |
| Timp1 | 5’-AACAGTGTTCAGGCTTCAGCTTT-3’ | 5’-GTGTGCACAGTGTTTCCCTGTT-3’ |
| sXBP1 | 5’-GAGTCCGCAGCAGGTG-3’ | 5’-GTGTCAGAGTCCATGGGA-3’ |
| GAPDH | 5’-AATGTGTCCGTCGTGGATCTGA-3’ | 5’-AGTGTAGCCCAAGATGCCCTTC-3’ |

Col1A1, collagen type I, α1; Col1A2, collagen type I, α2; Col3A1, collagen type III, α1; Col5A1, collagen type V, α1; Col5A2, collagen type V, α2; TGFβ, transforming growth factor β; MMP2, matrix metallopeptidase 2; MMP9, matrix metallopeptidase 9; Timp1, tissue inhibitor of metalloproteinase 1; sXBP1, spliced X-box binding protein; GAPDH, glyceraldehyde-3-phosphate dehydrogenase.
